# Nanostructure-Based Energy Electrocatalysis

**DOI:** 10.3390/nano16211409

**Published:** 2026-11-02

**Authors:** Xiangzhi Cui

**Affiliations:** State Key Laboratory of High Performance Ceramics, Shanghai Institute of Ceramics, Chinese Academy of Sciences, Shanghai 200050, China; cuixz@mail.sic.ac.cn

## 1. Introduction

The global transition toward carbon neutrality and sustainable energy systems has placed electrocatalysis at the center of modern energy research. Efficient electrocatalytic processes—including oxygen reduction reaction (ORR), oxygen evolution reaction (OER), hydrogen evolution reaction (HER), and small-molecule oxidation—are essential for the practical deployment of fuel cells, metal–air batteries, water electrolyzers, and electrochemical energy storage devices [1,2,3,4]. However, the large-scale application of these technologies remains limited by the sluggish kinetics of multi-electron transfer reactions, the insufficient long-term stability of catalysts under harsh operating conditions, and the reliance on costly noble-metal-based materials [5,6].

Nanostructured electrocatalysts offer a powerful platform to overcome these limitations. By precisely controlling size, morphology, composition, defect structure, and interfacial properties at the nanoscale, it is possible to tailor the electronic structure of active sites, maximize the density of accessible catalytic sites, and construct synergistic heterointerfaces that enhance both activity and durability [7,8,9]. Recent advances in carbon-based nanocomposites, layered double hydroxides (LDHs), metal–organic framework (MOF)-derived materials, transition metal phosphides, and MXene-supported hybrids have significantly expanded the design space for high-performance electrocatalysts [10,11,12]. At the same time, the field has increasingly recognized that the catalytic consequences of a given structural or electronic feature are highly reaction-specific, and that a deep understanding of structure–activity relationships requires the integration of advanced characterization, theoretical calculations, and device-level validation [13,14,15].

Collectively, the eight contributions in this Special Issue are not a random collection of nanomaterials for electrocatalysis. They converge on the central theme that nanoscale structure—including heterointerfaces, defects, coordination environments, alloying, and electrode architecture—governs charge transfer, intermediate adsorption, and durability in energy electrocatalysis. Together, they illustrate how nanostructure design can be used to optimize activity, selectivity, and stability for energy-related electrocatalytic reactions.

This Special Issue, “Nanostructure-Based Energy Electrocatalysis,” brings together eight original research articles that collectively address key challenges in the rational design of nanostructured electrocatalysts for energy conversion and storage. The contributions span fuel cell catalysts, bifunctional water-splitting electrocatalysts, chloride-ion battery cathodes, tribocatalytic nitrogen fixation, and supercapacitor electrodes, thereby providing a broad snapshot of the current state of the art and highlighting emerging research directions.

## 2. Overview of Contributions

Davletbaev et al. (Contribution 1) address the stability issue of carbon-supported Pt (Pt/C) catalysts for polymer electrolyte membrane fuel cells (PEMFCs). The authors designed a robust carbon shell encapsulated on Pt nanoparticles to suppress Pt dissolution, detachment, and agglomeration under harsh operating conditions. By systematically modulating the H_2_ content and flow rate of H_2_/N_2_ mixed gas during heat treatment, they demonstrated that the porosity of the carbon shell can be precisely tuned to facilitate oxygen adsorption for ORR while simultaneously acting as a protective barrier against catalyst degradation. This work provides valuable insights into the rational regulation of carbon shell layers for practical fuel cell applications.

Zou et al. (Contribution 2) report a NiTi-layered double hydroxide@carbon nanotube (NiTi-LDH@CNT) composite prepared via a reverse microemulsion method and applied for the first time as a cathode material for chloride-ion batteries (CIBs). The composite exhibits a high specific surface area of 266 m^2^ g^−1^ and a pore volume of 0.42 cm^3^ g^−1^, delivering a reversible specific capacity of 69 mAh g^−1^ after 150 cycles at 100 mA g^−1^, establishing NiTi-LDH@CNT as a promising cathode candidate for low-cost, high-safety CIBs. XPS and ex situ XRDEDS analyses reveal reversible Ni^2+^/Ni^3+^ and Ti^3+^/Ti^4+^ valence changes and reversible Cl^−^ migration during cycling.

Pei et al. (Contribution 3) construct an FeS and ZnS heterojunction on N,S-codoped carbon (FeZnS-SNC) derived from Zn– and Fe–organic frameworks via a facile one-step sulfonating strategy. XPS binding-energy shift analysis indicates electron transfer from FeS to adjacent ZnS at the heterointerfaces, which enables excellent ORR performance, with half-wave potentials of 0.94 V and 0.81 V in alkaline and acidic media, respectively, surpassing commercial 20 wt.% Pt/C. The flexible Zn–air battery equipped with FeZnS-SNC900 delivers a high open-circuit voltage of 1.45 V and a power density of 30.2 mW cm^−2^, and the assembled fuel cells achieve power outputs of 388.3 and 242.8 mW cm^−2^ under H_2_–O_2_ and H_2_–air conditions, respectively.

Fan et al. (Contribution 4) develop P-Fe_3_O_4_Fe@C electrodes with rich under-coordinated Fe atom interfaces derived from an iron-based metal–organic framework for pH-universal water splitting. XPS binding-energy analysis as well as the reference show that the introduction of under-coordinated Fe atoms increases the local charge density and polarizes the 3D orbital lone electrons, thereby promoting water adsorption and activation. The optimized catalyst exhibits low overpotentials of 160 mV and 214 mV at 10 mA cm^−2^ in acidic and alkaline electrolytes, respectively, along with good pH-universal long-term stability with little variation in half-wave potentials before and after 500 cycles, providing a new strategy for designing efficient pH-universal electrocatalysts.

Chen et al. (Contribution 5) report a bifunctional CoP_2_-Mo_4_P_3_NF electrocatalyst with rich heterointerfaces prepared by a two-step hydrothermal–phosphorylation method. The synergistic interaction between CoP_2_ and Mo_4_P_3_ heterogeneous interfaces optimizes the electronic structure of active sites, leading to weak H adsorption on Mo sites and increased redox activity of Co sites. The catalyst exhibits low overpotentials of 77.6 mV and 300.3 mV at 100 mA cm^−2^ for HER and OER, respectively, in 1.0 M KOH, and the assembled electrolyzer delivers 100 mA cm^−2^ at a cell voltage of 1.59 V while remaining stable for at least 370 h at 110 mA cm^−2^, demonstrating strong potential for practical water splitting.

Yang et al. (Contribution 6) prepare a self-supported FeNi LDHV_2_CTNF bifunctional electrode via hydrothermal deposition of FeNi LDH on a V_2_CT_x_NF substrate. XPS, XRD, and electron microscopy analyses confirm the strong interfacial interaction between V_2_CT_x_ and FeNi LDH effectively prevents LDH aggregation, exposes more catalytic active sites, and improves electrical conductivity and structural stability. The electrode requires overpotentials of only 222 mV and 151 mV for OER and HER at 10 mA cm^−2^ in 1 M KOH, respectively, and achieves overall water splitting at 1.74 V. This work offers a simple and effective strategy for constructing hierarchical electrocatalysts using conductive two-dimensional MXene substrates.

Wu et al. (Contribution 7) explore the tribocatalytic nitrogen fixation performance of two-dimensional graphitic carbon nitride (g-C_3_N_4_) by harvesting friction energy. At a stirring speed of 1000 r/min with methanol as a positive charge scavenger, the tribocatalytic NH_4_^+^ generation rate reaches 100.56 μmol L^−1^ g^−1^ h^−1^, which is 3.91 times higher than that without any scavenger. The tribocatalytic effect originates from charge transfer across the contact interface between the g-C_3_N_4_ catalyst and the stirring rod, enabling the conversion of N_2_ to ammonia. This work provides a potential avenue for harvesting mechanical energy from the natural environment for sustainable ammonia synthesis.

Gong et al. (Contribution 8) presents a facile strategy for tightly anchoring Co_3_O_4_ nanosheets onto carbon fiber conductive cloth (Co_3_O_4_@C) using a ZIF-67 sacrificial template via in situ impregnation and pyrolysis. The binder-free porous Co_3_O_4_@C electrode exhibits typical pseudocapacitance characteristics with a specific capacitance of ~251 F g^−1^ at 1 A g^−1^ and excellent cycling stability (90% retention after 5000 cycles at 3 A g^−1^). The assembled asymmetric flexible supercapacitor can drive a red LED for 6 min, demonstrating the potential of MOF-derived nanostructures for flexible energy storage devices.

## 3. Gaps and Future Directions

The contributions to this Special Issue collectively highlight several important gaps and future research directions in nanostructure-based energy electrocatalysis.

First, the need for reaction-specific descriptors and mechanistic understanding. The articles in this Issue demonstrate that the catalytic consequences of heterointerfaces, defects, and electronic structure modifications are highly dependent on the specific reaction and electrolyte environment. While descriptors such as the d-band center remain useful for certain reactions, their transferability across different reactions is limited. Future studies should focus on establishing descriptor sets that account for the specific adsorbates and elementary steps involved, supported by operando characterization techniques that can track the dynamic evolution of active sites under working conditions.

Second, the challenge of stability under industrial conditions. Several contributions in this Issue report impressive initial activity but relatively short stability tests (typically tens to hundreds of hours). For practical applications in fuel cells, water electrolyzers, and batteries, long-term durability under high current densities, corrosive electrolytes, and repeated cycling is essential. The design of catalysts with both high intrinsic activity and long-term durability—particularly in corrosive media such as seawater or acidic PEMFC environments—remains a critical challenge. Interfacial engineering strategies that decouple catalytic function from interfacial stability represent a promising direction.

Third, the integration of nanostructured catalysts into practical devices. Laboratory-scale half-cell measurements provide valuable mechanistic insights, but the translation of nanostructured electrocatalysts into membrane electrode assemblies and flow-cell electrolyzers requires careful consideration of mass transport, catalyst–membrane interfaces, and system-level engineering. Techno-economic analyses that evaluate the cost competitiveness of nanostructured catalysts relative to established benchmarks are essential for guiding practical development.

Fourth, the role of advanced characterization and theory. Operando and in situ techniques—including Raman, infrared, X-ray absorption spectroscopy, and scanning electrochemical microscopy—coupled with machine-learning-accelerated density functional theory calculations, are increasingly indispensable for resolving complex reaction mechanisms and identifying the true active sites under working conditions. The integration of these techniques with well-defined nanostructured model catalysts will accelerate the rational design of next-generation electrocatalysts.

Fifth, the expansion of electrocatalysis beyond conventional water splitting and ORR. The contributions in this Issue on tribocatalytic nitrogen fixation and chloride-ion batteries illustrate the growing breadth of nanostructure-based energy electrocatalysis. Future research should continue to explore unconventional reactions and energy storage mechanisms, such as electrosynthesis of value-added chemicals, nitrogen reduction, and beyond-Li-ion batteries, where nanostructured catalysts can offer unique advantages.

## 4. Conclusions

The Special Issue “Nanostructure-Based Energy Electrocatalysis” showcases the breadth and vitality of current research aimed at understanding and optimizing nanostructured electrocatalysts for sustainable energy conversion and storage. The eight contributions collected here span fuel cell catalysts, bifunctional water-splitting electrocatalysts, chloride-ion battery cathodes, tribocatalytic nitrogen fixation, and supercapacitor electrodes, collectively advancing our understanding of how nanoscale structure governs catalytic function. We thank all the authors, reviewers, and editorial staff whose efforts made this Special Issue possible. We hope that the articles presented here will stimulate further research and inspire new approaches to the design of efficient and durable electrocatalytic systems for a sustainable energy future.
